# Viral and Cellular Factors Contributing to the Hematogenous Dissemination of Human Cytomegalovirus via Polymorphonuclear Leukocytes

**DOI:** 10.3390/v14071561

**Published:** 2022-07-18

**Authors:** Berenike Braun, Kerstin Laib Sampaio, Anna K. Kuderna, Miriam Widmann, Christian Sinzger

**Affiliations:** Institute for Virology, Ulm University Medical Center, 89081 Ulm, Germany; kerstin.laib@uni-ulm.de (K.L.S.); anna.kuderna@uni-ulm.de (A.K.K.); miriam.hermann1@web.de (M.W.); christian.sinzger@uniklinik-ulm.de (C.S.)

**Keywords:** human cytomegalovirus, clinical isolates, hematogenous dissemination, herpesvirus entry, entry inhibitors

## Abstract

Polymorphonuclear leukocytes (PMNs) presumably transmit human cytomegalovirus (HCMV) between endothelial cells in blood vessels and thereby facilitate spread to peripheral organs. We aimed to identify viral components that contribute to PMN-mediated transmission and test the hypothesis that cellular adhesion molecules shield transmission sites from entry inhibitors. Stop codons were introduced into the genome of HCMV strain Merlin to delete pUL74 of the trimeric and pUL128 of the pentameric glycoprotein complex and the tegument proteins pp65 and pp71. Mutants were analyzed regarding virus uptake by PMNs and transfer of infection to endothelial cells. Cellular adhesion molecules were evaluated for their contribution to virus transmission using function-blocking antibodies, and hits were further analyzed regarding shielding against inhibitors of virus entry. The viral proteins pUL128, pp65, and pp71 were required for efficient PMN-mediated transmission, whereas pUL74 was dispensable. On the cellular side, the blocking of the αLβ2-integrin LFA-1 reduced virus transfer by 50% and allowed entry inhibitors to reduce it further by 30%. In conclusion, these data show that PMN-mediated transmission depends on the pentameric complex and an intact tegument and supports the idea of a virological synapse that promotes this dissemination mode both directly and via immune evasion.

## 1. Introduction

The human cytomegalovirus (HCMV) is widespread in the population, with an estimated seroprevalence of 83% worldwide [1]. Values vary widely by region, ranging from 45–66% in Europe to 90–100% in the Eastern Mediterranean or Africa [1,2]. After primary infection, which usually goes unnoticed in immunocompetent individuals [3], the virus remains in a latent state in the body for a lifetime [4]. HCMV can spread throughout the body via the bloodstream. In the blood, it is found predominantly in the cellular fraction [5,6] and disseminates via infected circulating endothelial cells, monocytes, or polymorphonuclear leukocytes (PMNs) [7,8,9]. This hematogenous spread is thought to facilitate the virus to rapidly infiltrate various tissues and organs. In HCMV-infected patients, high viral titers were determined, especially in PMNs [9,10]. These cells do not produce virus progeny themselves but rather act as vehicles by taking up the virus from infected cells and transferring it to other cells that are not yet infected [11].

To study this pathophysiologically relevant mode of dissemination in more detail, cell culture models that mimic the hematogenous spread of HCMV were developed. Uptake of HCMV in PMNs is accompanied by translocation of the viral tegument protein pp65 into the cell nucleus. This phenomenon, also exploited in the antigenemia assay in HCMV diagnostics [12], has already been repeatedly demonstrated and is also used as a surrogate marker in other in vitro models, such as induced pluripotent stem cell lines that develop into cerebral organoids [13,14,15]. To date, it is unclear whether pp65 is merely an indicator of virus uptake into PMNs or whether this protein has a functional role in the hematogenous dissemination of HCMV. Even less is known about the role of the tegument protein pp71 in PMN-mediated transfer, which is translated together with pp65 from a common bicistronic RNA and is important for infection of fibroblasts at low multiplicity of infection [16,17]. Remarkably, deletion of the homologous protein in rhesus cytomegalovirus (RhCMV) completely prevented host-to-host transmission by blood cells, whereas replication in fibroblasts was only slightly reduced which might indicate a particular role of pp71 in leukocytes [18]. With regard to the second step of the transfer, PMNs have previously been used as a vehicle to transmit a clinical HCMV strain to uninfected fibroblast cell cultures [19]. Recently, we established a model in which we used PMNs to transfer different clinical isolates and the laboratory strain Merlin from infected fibroblast cultures to uninfected fibroblasts, epithelial and endothelial cells [20]. Clinical isolates initially spread in a strictly cell-associated manner in cell culture [21,22] but start to release infectious progeny into the supernatant upon continued passaging. This phenotypic change is reflected by modifications in the viral genome, particularly in RL13 and the UL128 locus [22,23]. Obviously, the cell-free spreading mode provides an advantage in fibroblast culture resulting in the selection of either preexisting or de novo mutations that disrupt viral genes that would keep HCMV cell-associated [24]. The modified virus is then referred to as a laboratory strain. Strain Merlin has been cloned as a bacterial artificial chromosome (BAC), in which mutations in RL13 and UL128 were repaired to restore the wild-type genome. The introduction of a tetracycline operator allows this variant to grow strictly cell-associated without releasing cell-free infectious virus particles [24]. Thereby, this laboratory strain resembles clinical isolates in its spread. 

Such cell culture models can be used to study virus transmission at the molecular level, and they also allow evaluating potential inhibitors for their efficacy against this specific mode of dissemination. HCMV transfer to leukocytes appears to depend on the UL128 locus of the viral genome, which encodes for the three proteins pUL128, pUL130, and pUL131A [25]. Together with the glycoproteins gH and gL, they form the pentameric complex in the viral envelope, which also mediates entry into endothelial and epithelial cells [25,26,27,28]. Since this suggests similarities between virus uptake by leukocytes and entry into those cell types, the use of so-called entry inhibitors might also be considered against PMN-mediated spread of HCMV. While neuropilin-2 (Nrp2) has been identified as a cellular receptor of the pentameric complex on endothelial and epithelial cells [29], it is unclear whether it also contributes to pentamer-mediated uptake into leukocytes. The soluble decoy receptor Nrp2-Fc inhibits infection of endothelial and epithelial cells by binding to pUL128, pUL130, and pUL131A [29,30,31]. It is tempting to speculate whether this soluble receptor would also be effective during PMN-mediated transfer of HCMV to endothelial cells.

Entry inhibitors targeting the trimeric gH/gL/gO complex have already been analyzed for their effect on PMN-mediated transfer of HCMV. This complex binds to platelet-derived growth factor receptor alpha (PDGFRα) on the surface of fibroblasts and mediates entry of HCMV into this cell type [32,33,34]. A soluble derivative thereof, PDGFRα-Fc, can bind to cell-free virions and inhibit entry into fibroblasts, endothelial and epithelial cells, but it could not inhibit PMN-mediated transfer [20,30,33]. In contrast to the failure of these large decoy receptors, smaller peptide fragments derived from the extracellular PDGFRα domains 1–3 [33,35] reduced the efficiency of virus transmission by PMNs when added either during the initial uptake step or during the subsequent transfer step [20]. Similar phenomena are observed for the spread of clinical HCMV isolates in fibroblast cultures. While large entry inhibitors such as PDGFRα-Fc or neutralizing antibodies did not inhibit the strictly cell-associated growth of these viruses [35,36,37], PDGFRα-derived peptides reduced the number and size of infected foci [35]. The finding that large entry inhibitors fail to reduce cell-associated spread of HCMV isolates or transmission via PMNs, whereas smaller derivatives prove effective, also prompts considerations regarding the virus-cell interactions underlying this viral mode of transmission.

Cell-associated virus spread is thought to occur at close cell-cell contacts. However, a precise mechanism is still under discussion. There are different ideas for the transmission of virus particles between neighboring cells, for example, by retention and migration of particles on the cell surface of the productively infected cell until contact with neighboring cells, transmission in vesicles, or by polarized secretion [38,39]. A shared feature of these mechanisms is the involvement of adhesion molecules, which mediate contacts between neighboring cells. These molecules also play a central role in the concept of the so-called virological synapse, first described for human immunodeficiency virus 1 (HIV-1) transmission between monocyte-derived dendritic cells and T cells [40,41], but then proposed for other viruses as well [42,43]. Such a virological synapse might also form during PMN-mediated transfer of HCMV. For interaction between PMNs and endothelial cells in general, various adhesion molecules are known to be involved, including L-selectin, very late antigen-4 (VLA-4), or lymphocyte function-associated antigen 1 (LFA-1) on the surface of PMNs and E-selectin, vascular cell adhesion protein 1 (VCAM-1), or intercellular adhesion molecule 1 (ICAM-1) on endothelial cells [44,45]. Blocking of LFA-1 or ICAM-1 during the uptake step of HCMV from infected fibroblasts or endothelial cells was reported to reduce the number of pp65-positive PMNs [46], and blocking of these adhesion molecules during incubation of infected PMNs with uninfected fibroblasts was also reported to reduce virus transfer [19]. Together, the findings on the possible involvement of adhesion molecules in PMN-mediated transfer of HCMV and the inhibition of transfer exclusively by small entry inhibitors may indicate the formation of a virological synapse, whereby larger inhibitors such as neutralizing antibodies may fail to reach viral transmission by steric hindrance. 

Therefore, we aimed to apply our recently established transfer model to investigate whether (1) the viral tegument proteins pp65 and pp71 and the trimeric and pentameric glycoprotein complex are necessary for HCMV transmission via PMNs using stop mutants of the isolate-like strain Merlin and whether (2) large entry inhibitors like neutralizing antibodies can actually interfere with PMN-mediated transfer of HCMV isolates when the formation of the hypothetical synapse is disrupted by function-blocking antibodies against cellular adhesion molecules. These analyses might contribute to a better understanding and targeting of the pathophysiologically relevant hematogenous dissemination of HCMV.

## 2. Materials and Methods

### 2.1. Cells

Conditionally immortalized human endothelial cells (HEC-LTTs) [47,48] were cultivated in 0.1% gelatin (Sigma-Aldrich, St. Louis, MO, USA)-coated cell culture flasks in human endothelial cell growth medium (PromoCell, Heidelberg, Germany) supplemented with 2 μg/mL doxycycline (AppliChem, Darmstadt, Germany) and 100 µg/mL gentamicin (Sigma-Aldrich). For experiments, cells were seeded in medium without doxycycline. Primary human foreskin fibroblasts (HFFs) were cultivated in Dulbecco’s Modified Eagle Medium with GlutaMAX (Thermo Fisher Scientific, Waltham, MA, USA) supplemented with 10% fetal bovine serum (FBS; PAN Biotech, Aidenbach, Germany) and 100 µg/mL gentamicin (denoted as DMEM10).

Polymorphonuclear leukocytes (PMNs) were isolated from EDTA blood of HCMV-seronegative donors via Polymorphprep (Progen, Heidelberg, Germany) according to the manufacturer’s instructions. In addition, the remaining contaminating erythrocytes were lysed with NH_4_Cl for 5 min on ice. The PMNs were immediately used for the experiments.

### 2.2. Viruses

A Merlin bacterial artificial chromosome (BAC) clone that comprises the complete wild-type HCMV genome of strain Merlin [24] with a single tet operator upstream of the RL13 open reading frame [49] was used as the parental BAC for mutagenesis. For the generation of respective revertants, the introduced stop codons were removed in the mutant BACs and restored to the Merlin-RL13tetO wild-type nucleotide sequence. Mutant BACs Merlin-RL13tetO-UL74stop, Merlin-RL13tetO-UL128stop, and the revertant BAC Merlin-RL13tetO-UL74stopREV-UL128stopREV were described previously [49]. For the sake of clarity, this revertant was denoted as Merlin-RL13tetO-UL74stopREV in this manuscript. BAC-DNA was isolated using the NucleoBond Xtra Midi Kit (Macherey-Nagel, Düren, Germany), and the virus was reconstituted in HFFs by lipofection with the K2 Transfection System (Biontex Laboratories, München, Germany). Infected cells were cultured until 25 d post-transfection and then frozen into aliquots at −80 °C. From each culture, 15,000 cells were seeded into 96-well plates for 3 h, fixed, and stained for viral immediate early (IE) antigen (Ag)-positive cells to determine the individual infection rate for subsequent experiments.

Recent HCMV isolates were provided by the diagnostic laboratory of the Institute for Virology in Ulm and were propagated to infection rates of about 50–80% in HFFs. Until use in experiments, the infected cells were stored in aliquots at −80 °C. In each individual experiment, the isolates were analyzed for the cell-associated phenotype: The cell-free supernatant of the respective isolate was centrifuged at 2790× *g* for 10 min and incubated with 15,000 HFFs/well in a 96-well plate. After incubation overnight, the cells were fixed and stained for viral IE Ag via indirect immunofluorescence. An isolate was considered to grow in a strictly cell-associated manner if no more than 10 IE Ag-positive cells per well were detected. 

### 2.3. Generation of HCMV Mutants

Mutant BACs were generated using the markerless mutagenesis protocol developed by Tischer et al. [50] with primer sets as depicted in Table 1 or as described previously [49]. Plasmid pEP-Kan-S served as a template to obtain fragments that consisted of the 18-bp I-Sce I restriction site and a kanamycin resistance cassette flanked by repeated HCMV sequences containing homology to the desired site of insertion in the genome. A two-step PCR was performed as follows: In the first round of amplification, the respective long forward primer was used in combination with a short universal kanamycin reverse primer. After purification of products by Nucleospin PCR Clean-up Kit (Macherey Nagel, Düren, Germany), a second amplification was performed with the respective short forward and long reverse primers to obtain the final recombination fragment.

In the particular case of repairing the silent mutations of potential hairpin sites, a sequential amplification with several steps was applied, each with purification of products in between. For the recombination fragment of mutant repMerlin-UL83stop, an initial fragment was generated with primers *repMerlin-UL83stop for* and *Kanamycin universal rev*, followed by two consecutive rounds of amplification with primer pairs *Merlin-UL83stop short for* together with *repMerlin-UL83stop1 rev* and *Merlin-UL83stop short for* together with *repMerlin-UL83stop2 rev*. The generation of the recombination fragment for BAC repMerlin-UL83stopREV required four sequential rounds of amplification with the following primer pairs in the order listed: *repMerlin-UL83stopREV1 for* together with *Kanamycin universal rev*, *repMerlin-UL83stopREV2 for* together with *Kanamycin universal rev*, *Merlin-UL83stop short for* together with *repMerlin-UL83stopREV1 rev* and *Merlin-UL83stop short for* together with *repMerlin-UL83stopREV2 rev*.

The final products were again purified and then electroporated into recombination-activated GS1783 bacteria harboring either the Merlin-RL13tetO or the respective mutant BAC. After selection for kanamycin-resistant clones, all non-HCMV sequences were removed by intrabacterial I-Sce I digestion and a subsequent red recombination step. Each mutant was verified by restriction fragment length analysis, and Sanger sequencing and virus were reconstituted as described above. There was a report on the stability of HCMV BACs [51], in which random mutations were detected in only a few passages after reconstitution. To reduce the possibility that the effects on PMN-mediated transmission were due to such random unwanted side mutations, we used four fresh reconstitutions per mutant or revertant for our experiments.

### 2.4. PMN-Mediated Transmission of Cell-Associated HCMV Mutants

HFFs infected with Merlin-RL13tetO wild-type, mutants, or revertants in 24-well plates were used as donor cultures for PMN-mediated transmission. To equalize the number of infected cells in the different donor cultures, uninfected fibroblasts were added as required during seeding, and experiments were performed immediately the next day. Freshly isolated PMNs were incubated with the donor cultures at a 10:1 ratio for 3 h at 37 °C and then collected, taking care not to detach cells from the donor culture layer. One fraction of the collected PMNs was added at a ratio of 10:1 to uninfected HEC-LTTs seeded the day before on 96-well plates coated with 0.1% gelatin. These recipient cultures were incubated together with the PMNs for 3 h at 37 °C.

During this incubation, the donor cultures were fixed and stained for viral IE Ag by indirect immunofluorescence to precisely determine the number of infected donor cells. In addition, the remaining fraction of the collected PMNs was prepared for immunofluorescence staining by centrifugation in a StatSpin Cytofuge for 5 min at 395× *g* and drying for 1 h in front of a blower. These cytospot preparations served for quantification of pp65 uptake during the previous incubation of the PMNs with donor cultures.

After the 3 h incubation, PMNs were removed, and recipient cultures were incubated overnight. The next day, cells were fixed and stained for viral IE Ag or pp65 by indirect immunofluorescence. Nuclei were counterstained, and transfer efficiencies were determined as the ratio between the number of viral IE Ag- or pp65-positive nuclei and the total number of cells. The uptake/transfer efficiencies for each mutant/revertant were normalized to the infection rate of the respective donor culture to ensure that initial variation between donor cultures was eliminated and differences between viruses could be attributed solely to the PMN-mediated steps of the transmission.

### 2.5. Blocking of Cellular Adhesion Molecules during PMN-Mediated Transfer of HCMV Isolates

PMN-mediated transfer of clinical isolates in 6-well plates was conducted as described before with the following modification: After PMNs were collected from the donor cultures, they were preincubated for 30 min at 37 °C with function-blocking antibodies against the cellular adhesion molecules L-selectin, very late antigen-4 (VLA-4) or lymphocyte function-associated antigen 1 (LFA-1). Similarly, recipient cultures were preincubated for 30 min at 37 °C with antibodies against the adhesion molecules E-selectin, vascular cell adhesion protein 1 (VCAM-1), or intercellular adhesion molecule 1 (ICAM-1). Therefore, PMNs were either left untreated or were treated with 10 µg/mL anti-integrin αL (hu1124; Novus Biologicals, Littleton, CO, USA) or 20 µg/mL anti-integrin β2 (R&D Systems, Minneapolis, MN, USA) (both subunits of LFA-1), 5 µg/mL anti-integrin α4 (subunit of VLA-4, R&D Systems) or 25 µg/mL L-selectin (R&D Systems). HEC-LTTs were either left untreated or were treated with 25 µg/mL anti-E-selectin, 25 µg/mL anti-VCAM-1 or 10 µg/mL anti-ICAM-1 (all R&D Systems). Concentrations were chosen as described in the literature or by the manufacturer to induce inhibitory effects. PMNs and recipient cultures were then incubated as described, and cultures were incubated overnight before fixation and immunofluorescence staining.

### 2.6. Effects of HCMV Entry Inhibitors on PMN-Mediated Spread

The effect of HCMV entry inhibitors on PMN-mediated transfer of clinical isolates should be evaluated in comparison with or without preincubation of PMNs with anti-integrin β2. As described, PMNs were recollected from the donor cultures (clinical HCMV isolates) and were preincubated with 20 µg/mL anti-integrin β2 for 30 min at 37 °C or left untreated. This incubation was chosen as previous experiments showed that PMN-mediated transmission of HCMV was inhibited by 50% under these conditions. Thus, a sufficient number of recipient endothelial cells were infected to allow the detection of stronger inhibitory effects that would be caused by the entry inhibitors. HCMV-specific hyperimmunoglobulin (Cytotect, CP Biotest, Dreieich, Germany), recombinant PDGFRα-Fc and Nrp2-Fc (R&D Systems), and the PDGFRα-derived 30- and 40-mer peptides GD30, GT40 and IK40 [20,33,52] (Phtdpeptides, Shanghai, China) were added during incubation of either anti-integrin β2-treated or untreated PMNs with recipient HEC-LTTs at a concentration to achieve complete inhibition of cell-free virus (at least 10 × EC50). This resulted in concentrations of 0.5 mg/mL for hyperimmunoglobulin, 120 ng/mL for PDGFRα-Fc and 590 ng/mL for Nrp2-Fc. To exclude that the results are primarily determined by the amount of substance, the three PDGFRα-derived peptides were used at the same concentration of 0.45 mg/mL. A 0.4% dimethyl sulfoxide solution was used as an untreated control for the peptide dilutions, corresponding to the concentration of this solvent in the peptide treatments. For the other antiviral agents, DMEM10 was used as untreated control. The transfer efficiencies in the presence of HCMV inhibitors without anti-integrin β2 treatment were then normalized to the untreated controls. Transfer efficiencies in the presence of inhibitors and anti-integrin β2 were normalized to anti-integrin β2 treatment only.

### 2.7. Indirect Immunofluorescence

HFFs and HEC-LTTs were fixed for 5 min at room temperature (RT) with 80% acetone. Viral IE Ag was detected by incubation with monoclonal mouse antibody CH160 (antibodies-online Inc., Limerick, PA, USA) for 90 min and incubation with Cy3-goat-anti-mouse Ig F(ab’)_2_ (Jackson ImmunoResearch, West Grove, PA, USA) for 60 min at 37 °C. Staining revealed a red nuclear fluorescence pattern. When viral pp65 was detected using monoclonal mouse antibody 28–77 ([53] kindly provided by W. Britt, Birmingham, AL, USA) for 90 min and Cy3-goat-anti-mouse Ig F(ab’)_2_ for 60 min at 37 °C, IE Ag was instead detected by incubation with polyclonal rabbit antiserum against IE2-p86 [54] (kindly provided by T. Stamminger, Ulm, Germany) for 90 min and Alexa488-goat-anti-rabbit IgG H&L for 60 min. Staining revealed a red nuclear fluorescence pattern for pp65 and a green one for IE Ag. The nuclei were counterstained with 4′,6-diamidino-2-phenylindole (DAPI; Sigma-Aldrich) for 8 min at RT. Cells were washed with phosphate-buffered saline (PBS) after each incubation step. 

Cytocentrifuged PMN preparations were fixed with 1% paraformaldehyde (Sigma-Aldrich) for 10 min at RT, permeabilized with 10% sucrose (Sigma-Aldrich), 1% FBS, and 0.5% Nonidet P40 (Sigma-Aldrich) for 10 min at RT and then blocked with 5% FBS for 30 min at 37 °C. Viral pp65 was detected using monoclonal mouse antibody 28-77 and Alexa488-goat-anti-mouse Ig F(ab’)_2_ (Thermo Fisher Scientific, Waltham, MA, USA). Staining resulted in a green nuclear fluorescence signal. Nuclei were counterstained with DAPI for 30 s at RT. PMNs were washed with PBS after each incubation step.

### 2.8. Immunoblotting

Cells were harvested and lysed for 10 min at 95 °C in lysis sample buffer (0.5 M Tris HCl pH 6.8, 10% glycerin, 2% sodium dodecyl sulfate, 5% β-mercaptoethanol, 0.05% bromphenol blue). Lysates were loaded onto 10% polyacrylamide gels, and electrophoresis was performed. Proteins were then transferred onto PVDF membranes (Bio-Rad, Hercules, CA, USA) in Trans-Blot Turbo Transfer Buffer (Bio-Rad). Membranes were blocked with 5% milk powder in PBS + 0.1% Tween for 1 h at RT and incubated overnight at 4 °C with primary antibodies against viral pp71 (2H10-9 [55], kindly provided by Giada Frascaroli and Wolfram Brune, Hamburg, Germany), pp65 (28–77 [53], kindly provided by W. Britt), IE1 (63–27 [56], kindly provided by W. Britt), MCP (28–4 [57], kindly provided by W. Britt) and cellular β-actin (AC-15; Sigma-Aldrich). Membranes were washed with PBS + 0.1% Tween and incubated for 1 h at RT with secondary antibody horseradish peroxidase-conjugated goat IgG anti-mouse IgG (H + L) (Dianova, Hamburg, Germany). After washing, chemiluminescence detection and quantification were performed using Fusion FX Spectra (Vilber Lourmat Deutschland GmbH, Eberhardzell, Germany) and Fusion SL (Peqlab, Erlangen, Germany) imaging systems.

### 2.9. Statistical Analysis

Data sets were analyzed with a one-way ANOVA for differences between the different groups. When significant differences were found, appropriate post hoc analysis was performed. Depending on whether the data set was balanced or not, either Bonferroni’s comparison test or Fisher’s LSD test was chosen. *p*-values < 0.05 were considered marginally significant, <0.01 significant, and <0.001 highly significant.

## 3. Results

Recently, we have established a cell culture model in which we used cell-associated HCMV isolates as donor cultures and transferred them to uninfected fibroblast, epithelial, and endothelial recipient cultures using leukocytes as vehicles [20]. In the present study, we wanted to apply this model to identify viral proteins critical for PMN-mediated hematogenous dissemination and to examine the hypothesis that cellular adhesion molecules form a reaction space between PMN and endothelial cell, a so-called virological synapse, which promotes virus transfer by shielding transmission sites against neutralizing antibodies.

Although hematogenous spread in the infected host might be best simulated by using endothelial cells both as donor cultures and recipient cultures for PMN-mediated transmission, we preferred to use infected fibroblasts as donor cultures for practical reasons. Focal spread of HCMV isolates was less efficient in endothelial cells as compared to fibroblasts [20], and the uptake of virus into PMNs was hence more reliable and easier to standardize if fibroblasts were used. This was an important prerequisite for this study that was more focused on the subsequent transfer step from infected PMNs to uninfected endothelial cells.

### 3.1. Generation of HCMV Mutants and Revertants Thereof

The trimeric and the pentameric glycoprotein complex of HCMV govern the entry into various cell types such as fibroblasts, endothelial and epithelial cells. We aimed to investigate their contribution to PMN-mediated uptake and transfer of cell-associated HCMV. Representing the pentameric complex, we focused on pUL128 as one of its three accessory proteins. The impact of pUL128 and the other accessory pentamer proteins, pUL130 and pUL131A, on the uptake of HCMV in PMNs have been studied before using knockout mutants in the background of another HCMV strain. This demonstrated their importance, as the virus was not taken up into PMNs if any of the three genes were mutated [25]. However, the subsequent step of virus transfer to endothelial cells remains to be investigated. Studies with regard to the contribution of the trimeric glycoprotein complex of HCMV to uptake and transfer via PMNs are lacking so far. Here, we focused on pUL74 to represent the trimeric complex. While uptake of the tegument protein pp65 into PMNs is commonly used as a diagnostic marker for HCMV infection, it is not yet known whether this protein is actually required for virus uptake into these cells and transfer to endothelial cells. The gene encoding pp65, UL83, is reported to be transcribed into a bicistronic RNA together with the upstream gene UL82 [16], which encodes the tegument protein pp71. Given this shared transcription unit, it is reasonable to speculate that if pp65 contributes to PMN-mediated spread of HCMV, pp71 may also be involved, and recent data from an animal model support this assumption to some extent [18]. 

**Figure 1:**
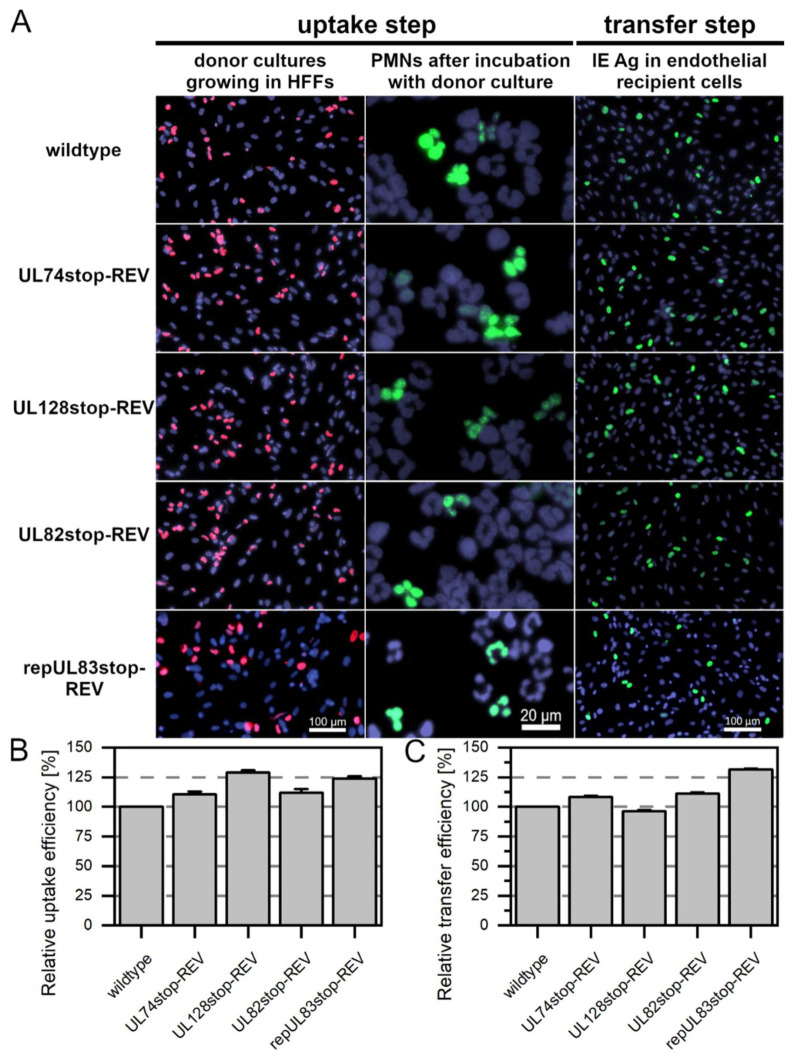
Comparison of Merlin-RL13tetO wild-type and the revertants used in this study during PMN-mediated transmission. (**A**) PMNs were isolated from EDTA blood of HCMV-seronegative donors and incubated for 3 h at 37 °C with Merlin-RL13tetO, Merlin-RL13tetO-UL74stop-REV, Merlin-RL13tetO-UL128stop-REV, Merlin-RL13tetO-UL82stop-REV or repMerlin-RL13tetO-UL83stop-REV. PMNs were recollected, and a fraction was used for the preparation of cytospots. Donor cultures were fixed and stained via indirect immunofluorescence for viral IE Ag (pink nuclei). PMNs were fixed and stained for viral pp65 (green nuclei). The remaining PMNs were incubated with uninfected recipient HEC-LTTs for 3 h at 37 °C. After incubation, PMNs were removed. On the next day, cultures were fixed and stained for viral IE Ag via indirect immunofluorescence (green nuclei). Cell nuclei were counterstained with DAPI (purple nuclei). (**B**) The uptake efficiency was calculated as the fraction of pp65-positive PMNs compared to all PMNs and was normalized to the uptake of wild-type Merlin-RL13tetO. Bars indicate mean values of four individual experiments, and error bars represent the standard error of the mean (SEM). (**C**) The transfer efficiency was calculated as the fraction of IE Ag-positive cells compared to the overall cell count and was normalized to PMN-mediated transfer of wild-type Merlin-RL13tetO. Bars indicate mean values of four individual experiments, and error bars represent the SEM.

We have knocked out the genes of interest, UL128, UL74, UL83, and UL82, in the BAC Merlin-RL13tetO by introducing two stop codons in each gene via markerless mutagenesis [50]. To control for the effects of unwanted side mutations, a revertant was generated for each stop mutant, using the mutant BAC as the parental sequence and exchanging the introduced stop codons for the original wild-type nucleotide sequence. The initial aim was to assess whether the four revertants behaved similarly to the wild-type Merlin-RL13tetO regarding PMN-mediated uptake and transfer to endothelial cells. PMNs were freshly isolated from EDTA blood of HCMV-seronegative donors by density centrifugation, collected from the gradient, and immediately applied to the donor cultures for 3 h at 37 °C. After incubation with wild-type Merlin or the revertants, PMNs were applied to uninfected HEC-LTT recipient cultures for 3 h at 37 °C. Aliquots were used to prepare cytospots that were stained for viral pp65 to determine how efficient the uptake of virus into PMNs was (Figure 1). As automatic counting of PMN nuclei was not feasible, the pp65-positive area was compared to the overall nuclear area. PMNs were removed, and the recipient cultures were incubated overnight. The next day, HEC-LTTs were fixed and stained for viral IE Ag via indirect immunofluorescence. Nuclei were counterstained with DAPI. To determine how efficiently the virus was transferred from the PMNs to endothelial cells, transfer efficiencies were calculated as the percentage of IE Ag-positive cells compared with the total cell number and normalized to wild-type transfer (Figure 1).

Viral pp65 was detected in about 5% of the PMNs that had been incubated with wild-type virus or the revertants Merlin-RL13tetO-UL74stop-REV, Merlin-RL13tetO-UL128stop-REV, and Merlin-RL13tetO-UL82stop-REV. The transfer step of HCMV from the infected PMNs to the endothelial cells was also similar for wild-type Merlin-RL13tetO and these three revertants. In initial experiments, the uptake of virus from Merlin-RL13tetO-UL83stop-REV to PMNs was significantly reduced by almost 75% compared to the wild-type (Supplementary Figure S1). The subsequent step of transfer from infected PMNs to endothelial cells was significantly reduced by 98% compared to the wild-type. This was surprising, as Merlin-RL13tetO-UL83stop-REV grew like wild-type virus in fibroblast cultures, excluding a general replication defect (Supplementary Figure S2). The only genetic difference between the two viruses are silent mutations in codons 9, 15, and 17 that appeared inevitable during the introduction of the stop mutations to avoid deleterious hairpin formation by the respective primers. Whole genome sequencing confirmed the absence of any other second site mutation (Supplementary Table S1).

As our initial strategy had failed, in that the phenotype of Merlin-RL13tetO-UL83stop-REV was not completely restored to wild-type levels, we made an additional attempt to repair the silent mutations in this revertant to generate a viral genome identical to that of the wild-type. Using Merlin-RL13tetO-UL83stop-REV as the parental BAC, the silent mutations in codons 9, 15, and 17 were repaired to the original nucleotide sequence as in Merlin-RL13tetO wild-type. For that, the mutagenesis protocol after Tischer et al. was modified by sequential amplification with several steps in order to generate the recombination fragment. When using cells infected with the reconstituted virus repMerlin-RL13tetO-UL83stop-REV as a donor culture, virus uptake and transfer were not altered in comparison to the wild-type (Figure 1), as expected. A similar cloning strategy was then applied to Merlin-RL13tetO-UL83stop to repair the silent mutation also in this background and generate a mutant that differed from wild-type and revertant only by the two introduced stop codons at positions 11 and 16. The following experiments were hence conducted with repMerlin-RL13tetO-UL83stop-REV and repMerlin-RL13tetO-UL83stop. Each mutant was compared to the respective revertant throughout the study. 

### 3.2. While pUL128 Is Essential for Uptake of HCMV by PMNs, Glycoprotein O Is Dispensable for Uptake and Transfer

To investigate the contribution of the pentameric and trimeric glycoprotein complex to PMN-mediated dissemination of HCMV, Merlin-RL13tetO-UL128stop, and Merlin-RL13tetO-UL74stop or their respective revertants were used as donor cultures. The transfer was performed as described in the previous section. The uptake and transfer efficiencies of the mutants were normalized to those of the respective revertants.

**Figure 2:**
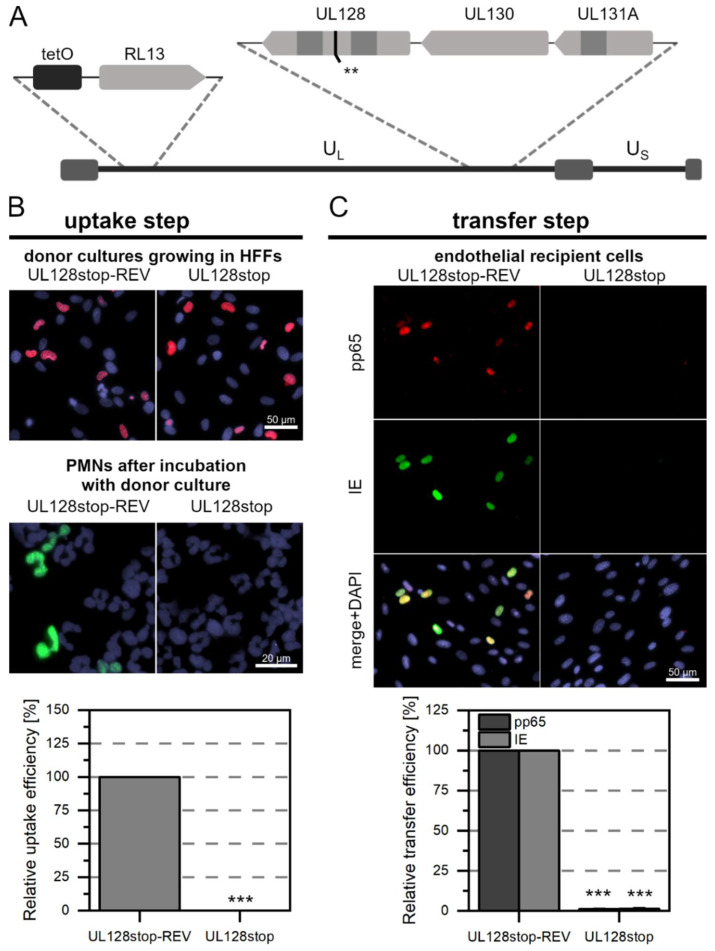
The pentameric HCMV complex is essential for PMN-mediated transmission to endothelial cells as it is critical for virus uptake into PMNs. (**A**) Genomic overview of the generated Merlin mutant representing the unique long (U_L_) and short (U_S_) sections: the mutant carries the tet operator upstream of RL13. In comparison to the wild-type, two consecutive stop codons (**) were introduced into the ORF of the UL128 gene of the UL128-131A locus, as indicated. (**B**) Characterization of the uptake step to PMNs. PMNs were isolated from EDTA blood of HCMV-seronegative donors and incubated for 3 h at 37 °C with Merlin-RL13tetO-UL128stop or its revertant Merlin-RL13tetO-UL128stop-REV. PMNs were recollected, and a fraction was used for the preparation of cytospots. Donor cultures were fixed and stained via indirect immunofluorescence for viral IE Ag (pink nuclei). PMNs were fixed and stained for viral pp65 (green nuclei). Nuclei were counterstained with DAPI (purple nuclei). The uptake efficiency was calculated as the fraction of pp65-positive PMNs compared to all PMNs and is shown relative to that of the revertant. Bars indicate mean values of four individual experiments, and error bars represent the standard error of the mean (SEM). Asterisks indicate significant differences as compared to the revertant (*** *p*-value < 0.001). (**C**) Characterization of the transfer step from PMNs to recipient endothelial cells. The remaining PMNs were incubated with uninfected recipient HEC-LTTs for 3 h at 37 °C. After incubation, PMNs were removed. On the next day, cultures were fixed and stained for viral IE Ag (green nuclei) and pp65 (red nuclei) via indirect immunofluorescence. Cell nuclei were counterstained with DAPI (purple nuclei). The transfer efficiency was calculated as the fraction of pp65- or IE Ag-positive cells compared to the overall cell count and is shown relative to that of the revertant. Bars indicate mean values of 4 individual experiments, the error bars represent the SEM. Asterisks indicate significant differences as compared to the revertant (*** *p*-value < 0.001).

**Figure 3:**
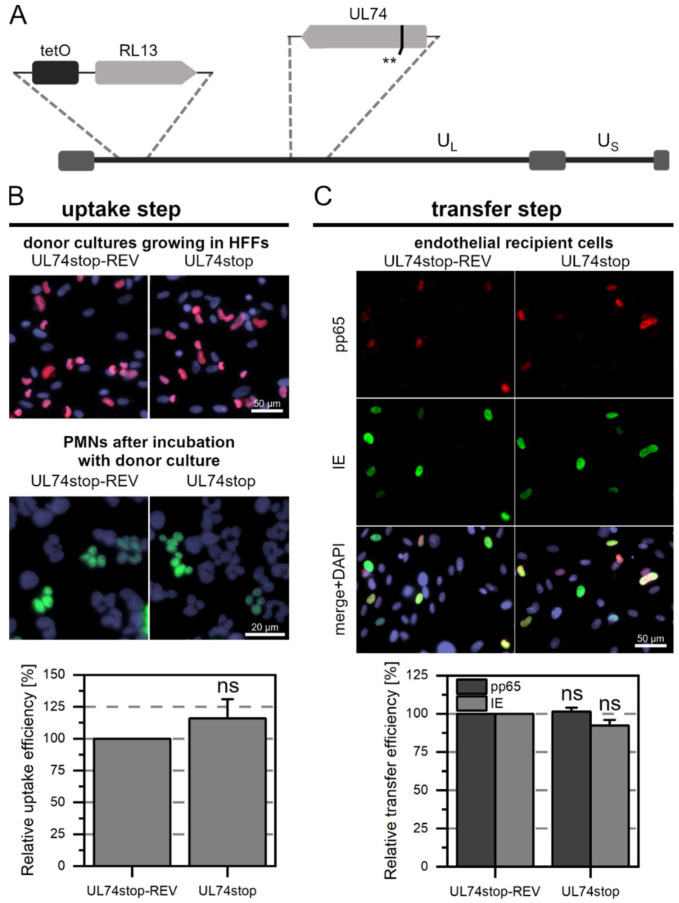
The trimeric complex of HCMV is dispensable for uptake into PMNs and subsequent transfer to endothelial cells. (**A**) Genomic overview of the generated Merlin mutant representing the unique long (U_L_) and short (U_S_) sections: the mutant carries the tet operator upstream of RL13. In comparison to the wild-type, two consecutive stop codons (**) were introduced into the ORF of UL74 as indicated. (**B**) Characterization of the uptake step to PMNs. PMNs were isolated from EDTA blood of HCMV-seronegative donors and incubated for 3 h at 37 °C with Merlin-RL13tetO-UL74stop or its revertant Merlin-RL13tetO-UL74stop-REV. PMNs were recollected, and a fraction was used for the preparation of cytospots. Donor cultures were fixed and stained via indirect immunofluorescence for viral IE Ag (pink nuclei). PMNs were fixed and stained for viral pp65 (green nuclei). Nuclei were counterstained with DAPI (purple nuclei). The uptake efficiency was calculated as the fraction of pp65-positive PMNs compared to all PMNs and is shown relative to that of the revertant. Bars indicate mean values of four individual experiments, the error bars represent the standard error of the mean (SEM). Ns indicates that were no significant differences as compared to the revertant (*p*-value > 0.05). (**C**) Characterization of the transfer step from PMNs to recipient endothelial cells. The remaining PMNs were incubated with uninfected recipient HEC-LTTs for 3 h at 37 °C. After incubation, PMNs were removed. On the next day, cultures were fixed and stained for viral IE Ag (green nuclei) and pp65 (red nuclei) via indirect immunofluorescence. Cell nuclei were counterstained with DAPI (purple nuclei). The transfer efficiency was calculated as the fraction of pp65- or IE Ag-positive cells compared to the overall cell count and is shown relative to that of the revertant. Bars indicate mean values of four individual experiments, the error bars represent the SEM. Ns indicates that were no significant differences as compared to the revertant (*p*-values > 0.05).

Pp65 was not detected in any of the PMNs that were incubated with Merlin-RL13tetO-UL128stop (Figure 2A). In contrast, about 5% of the PMNs that were incubated with Merlin-RL13tetO-UL128stop-REV were pp65-positive (Figure 2B). This difference was highly significant (*p*-value < 0.001). Consistent with the absence of pp65 in PMNs after incubation with Merlin-RL13tetO-UL128stop, no pp65 was detected in endothelial cells after incubation with these PMNs. This contrasted again with the revertant, where numerous pp65-positive and IE Ag-positive cells were found. As expected from the complete lack of detectable pp65-uptake in the first step with the stop mutant, the number of infected cells in the recipient cultures was significantly reduced by 99% (*p*-values < 0.001, Figure 2C). After incubation with Merlin-RL13tetO-UL74stop (Figure 3A) or Merlin-RL13tetO-UL74stop-REV, about 5% of PMNs were pp65-positive (Figure 3B). There was no significant difference in the uptake efficiency between mutant and revertant. The virus transfer step from PMNs to endothelial cells also resulted in similar transfer efficiencies: The fraction of pp65 or IE Ag-positive cells was similar for mutant and revertant and did not show significant differences (Figure 3C). Taken together, these results support the idea that the pentameric complex is necessary for uptake of HCMV into PMNs and hence for transmission to uninfected endothelial cells. In contrast, the trimeric complex seems to be neglectable for this mode of virus dissemination.

### 3.3. The Tegument Proteins pp65 and pp71 Contribute to PMN-Mediated Transmission of HCMV

It is unknown whether pp65 or pp71 are required for efficient uptake and transfer of HCMV via PMNs. To investigate their impact on this mode of spread, we used repMerlin-RL13tetO-UL83stop and Merlin-RL13tetO-UL82stop or their respective revertants as donor cultures. The mutants were analyzed regarding the expression of pp65 and pp71 via quantitative western blot analyses with cells from four independent virus reconstitutions (Supplementary Figure S3). As expected, the pp65-specific band was missing in repMerlin-RL13tetO-UL83stop, which was also reflected in an almost complete reduction in the signal intensity in the quantitative analysis. Similarly, the pp71-specific band was absent in Merlin-RL13tetO-UL82stop, and the signal intensity was significantly reduced compared with Merlin-RL13tetO-UL82stop-REV and Merlin-RL13tetO-wild type. The residual intensity of the pUL82stop mutant in the analyses with the anti-pp71 antibody is likely due to an additional band detected slightly below the pp71-specific band. This lower nonspecific band is most probably due to pp65, as it is not detected in repMerlin-RL13tetO-UL83stop. 

**Figure 4:**
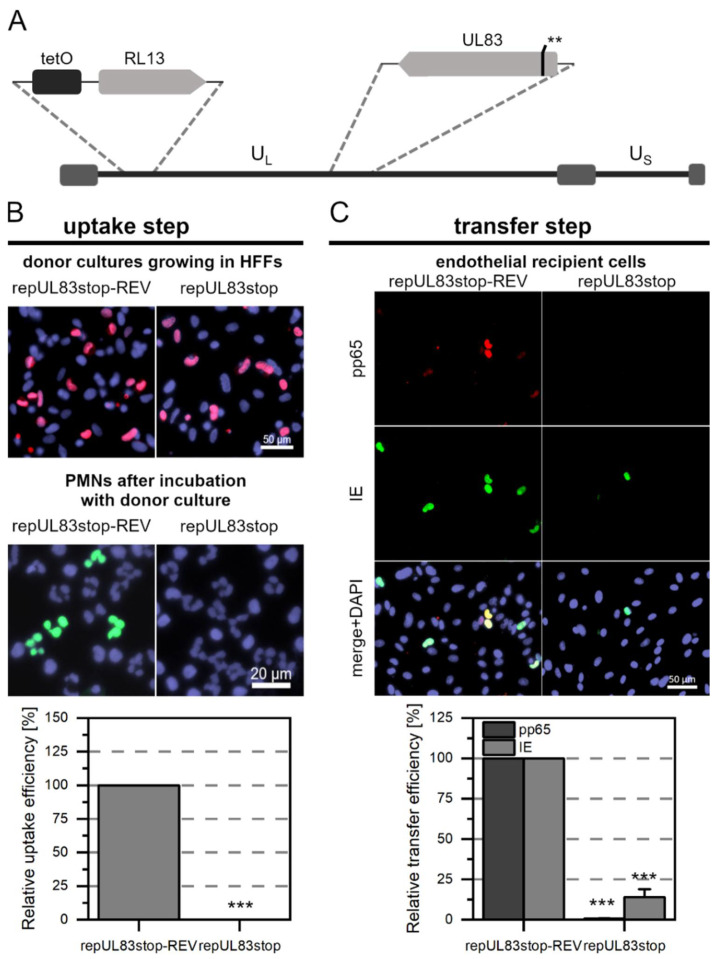
The tegument protein pp65 of HCMV is required for PMN-mediated transfer to endothelial cells. (**A**) Genomic overview of the generated Merlin mutant representing the unique long (U_L_) and short (U_S_) sections: the mutant carries the tet operator upstream of RL13. In comparison to the wild-type, two consecutive stop codons (**) were introduced into the ORF of UL83 as indicated. (**B**) Characterization of the uptake step to PMNs. PMNs were isolated from EDTA blood of HCMV-seronegative donors and incubated for 3 h at 37 °C with repMerlin-RL13tetO-UL83stop or its revertant repMerlin-RL13tetO-UL83stop-REV. PMNs were recollected, and a fraction was used for the preparation of cytospots. Donor cultures were fixed and stained via indirect immunofluorescence for viral IE Ag (pink nuclei). PMNs were fixed and stained for viral pp65 (green nuclei). Nuclei were counterstained with DAPI (purple nuclei). The uptake efficiency was calculated as the fraction of pp65-positive PMNs compared to all PMNs and is shown relative to that of the revertant. Bars indicate mean values of four individual experiments, and the error bars represent the standard error of the mean (SEM). Asterisks indicate significant differences as compared to the revertant (*** *p*-value < 0.001). (**C**) Characterization of the transfer step from PMNs to recipient endothelial cells. The remaining PMNs were incubated with uninfected recipient HEC-LTTs for 3 h at 37 °C. After incubation, PMNs were removed. On the next day, cultures were fixed and stained for viral IE Ag (green nuclei) and pp65 (red nuclei) via indirect immunofluorescence. Cell nuclei were counterstained with DAPI (purple nuclei). The transfer efficiency was calculated as the fraction of pp65- or IE Ag-positive cells compared to the overall cell count and is shown relative to that of the revertant. Bars indicate mean values of four individual experiments, and the error bars represent the SEM. Asterisks indicate significant differences as compared to the revertant (*** *p*-value < 0.001).

**Figure 5:**
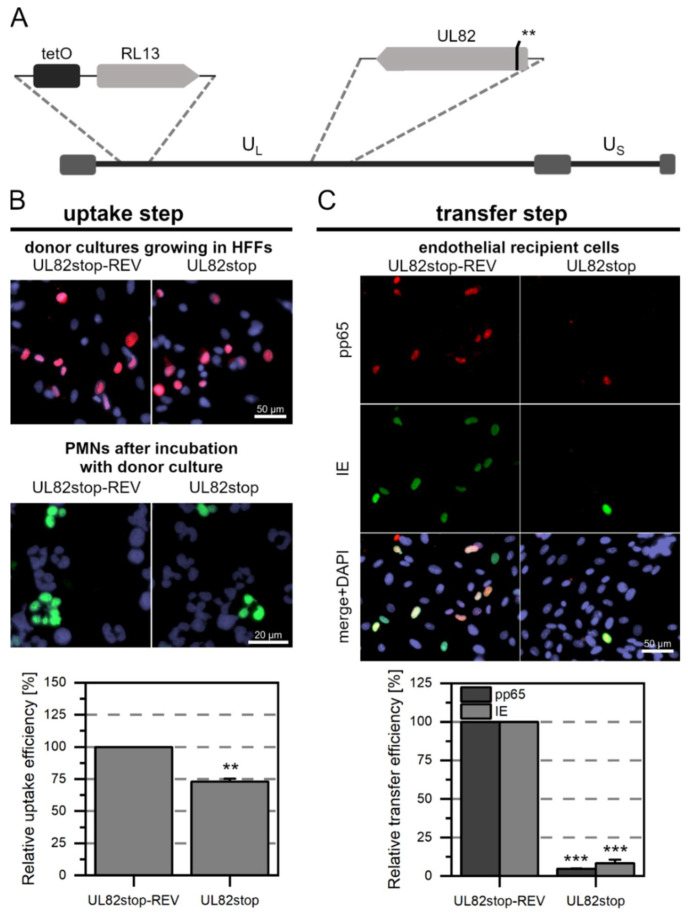
The tegument protein pp71 of HCMV is required for uptake into PMNs and subsequent transfer to endothelial cells. (**A**) Genomic overview of the generated Merlin mutant representing the unique long (U_L_) and short (U_S_) sections: the mutant carries the tet operator upstream of RL13. In comparison to the wild-type, two consecutive stop codons (**) were introduced into the ORF of UL82 as indicated. (**B**) Characterization of the uptake step to PMNs. PMNs were isolated from EDTA blood of HCMV-seronegative donors and incubated for 3 h at 37 °C with Merlin-RL13tetO-UL82stop or its revertant Merlin-RL13tetO-UL82stop-REV. PMNs were recollected, and a fraction was used for preparation of cytospots. Donor cultures were fixed and stained via indirect immunofluorescence for viral IE Ag (pink nuclei). PMNs were fixed and stained for viral pp65 (green nuclei). Nuclei were counterstained with DAPI (purple nuclei). The uptake efficiency was calculated as the fraction of pp65-positive PMNs compared to all PMNs and is shown relative to that of the revertant. Bars indicate mean values of four individual experiments, the error bars represent the standard error of the mean (SEM). Asterisks indicate significant differences as compared to the revertant (** *p*-value < 0.01). (**C**) Characterization of the transfer step from PMNs to recipient endothelial cells. The remaining PMNs were incubated with uninfected recipient HEC-LTTs for 3 h at 37 °C. After incubation, PMNs were removed. On the next day, cultures were fixed and stained for viral IE Ag (green nuclei) and pp65 (red nuclei) via indirect immunofluorescence. Cell nuclei were counterstained with DAPI (purple nuclei). The transfer efficiency was calculated as the fraction of pp65- or IE Ag-positive cells compared to the overall cell count and is shown relative to that of the revertant. Bars indicate mean values of four individual experiments, and the error bars represent the SEM. Asterisks indicate significant differences as compared to the revertant (*** *p*-value < 0.001).

The experimental procedure to investigate the contribution of pp65 and pp71 to PMN-mediated transmission of HCMV was performed as described in the previous sections. As expected, we did not detect fluorescence signals in PMNs after incubation with repMerlin-RL13tetO-UL83stop (Figure 4A) (*p*-value < 0.001), whereas pp65-positive PMNs were detectable after incubation with the revertant repMerlin-RL13tetO-UL83stop-REV (Figure 4B). Similarly, pp65-positive nuclei could not be detected in the endothelial cells after incubation with the PMNs for repMerlin-RL13tetO-UL83stop (*p*-value < 0.001), but for the revertant (Figure 4C). In contrast, IE Ag-positive cells were also detected in the recipient cells after incubation with the mutant-treated PMNs. However, compared to the revertant, the fraction was significantly reduced to only 14% (*p*-value < 0.001). After incubation with Merlin-RL13tetO-UL82stop (Figure 5A) or Merlin-RL13tetO-UL82stop-REV, pp65-positive PMNs were detected in the cytospots (Figure 5B). In comparison to the revertant, the number was significantly reduced by 28% for PMNs that had been incubated with the mutant (*p*-value < 0.01). The transfer to endothelial cells was also less efficient: The transfer efficiency measured as the fraction of IE Ag-positive cells was significantly reduced by 91% (*p* values < 0.001, Figure 5C). Pp71 is a major regulator of viral gene expression [58]. It counteracts intrinsic cellular factors such as Daxx. The use of an HCMV mutant lacking pp71 might bias the transfer efficiency measured by IE Ag expression, as the transactivating role of pp71 in viral gene expression would not be given. Therefore, detection of pp65 in recipient endothelial cells was particularly important as a surrogate marker for evaluating the effects of Merlin-RL13tetO-UL82stop on the transfer step. Transfer of mutant-infected PMNs to endothelial cells, as measured by pp65-positive recipient cells, was reduced by 95% compared with the revertant (*p*-values < 0.001, Figure 5C). Thus, the mutant showed a similar effect on transfer efficiency compared with the revertant for both readouts.

In summary, the PMN-mediated transmission was significantly reduced for both tegument protein mutants compared with the respective revertant. These inhibitory effects suggest that pp65 and pp71 are required for efficient dissemination of HCMV via leukocytes.

### 3.4. LFA-1 Is Required for Efficient Transfer of Cell-Associated HCMV Isolates via PMNs

**Figure 6:**
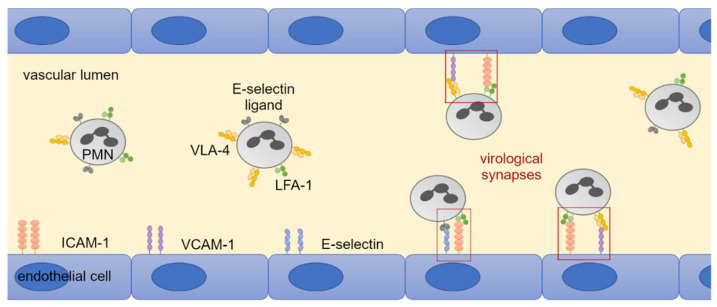
Hypothetical scheme of binding of PMNs to endothelial cells in the vascular lumen with the formation of virological synapses. PMNs can bind to receptors on endothelial cells such as ICAM-1, VCAM-1, and E-selectin via adhesion molecules such as LFA-1, VLA-4, and E-selectin ligand. The binding creates a spatial proximity between PMN and endothelial cells. This reaction space can serve for virus transmission and is often referred to as the virological synapse by analogy with the immunological synapse. Cell and receptor size as well as expression ratios are not shown realistically but are simplified to highlight predicted interactions.

**Figure 7:**
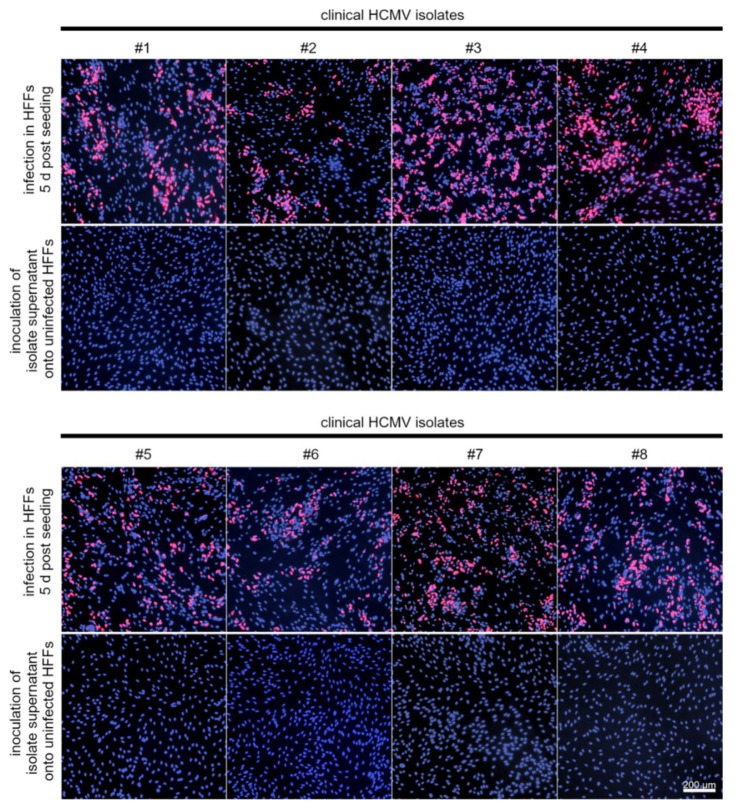
Eight different cell-associated HCMV isolates were used as donor cultures for PMN-mediated transmission experiments. Uninfected HFFs were co-cultured with HFFs infected with eight different clinical isolates. These cultures were used as donor cultures for transfer experiments 5 d post-seeding (the respective upper panels). The cell culture supernatant of each isolate was examined on the day of the individual experiment for cell-free infectivity in uninfected HFFs (the respective lower panels). Cells were fixed on the next day and stained via indirect immunofluorescence for viral IE Ag (pink nuclei). Cell nuclei were counterstained with DAPI (purple nuclei).

**Figure 8:**
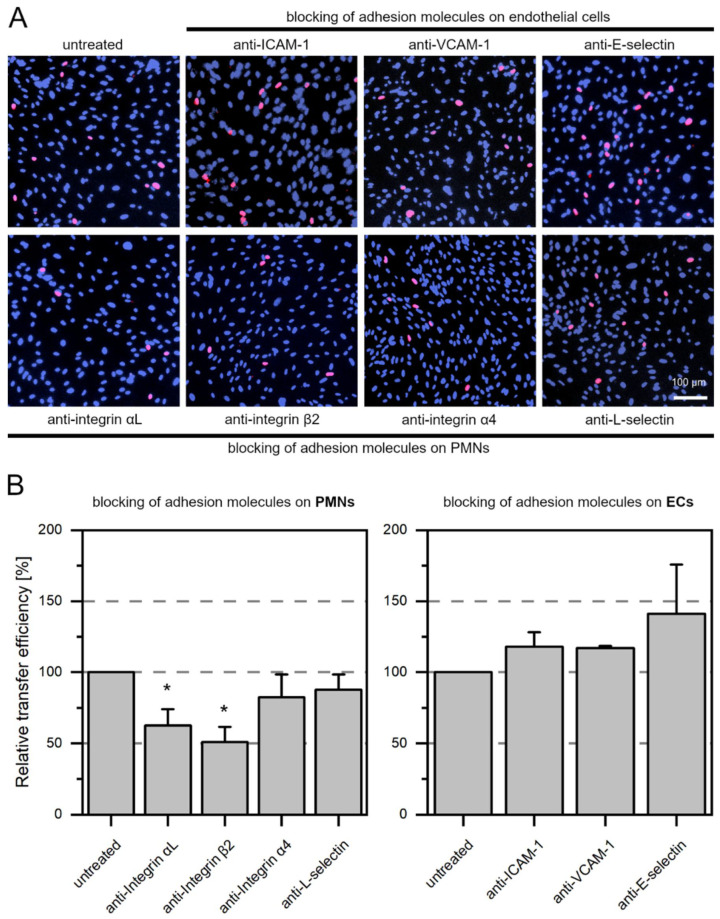
Blocking of LFA-1 subunits αL or β2 reduces the efficiency of HCMV transfer via PMNs. PMNs were isolated from EDTA blood of HCMV-seronegative donors and incubated for 3 h at 37 °C with clinical isolates. PMNs were recollected and either left untreated or were treated for 30 min at 37 °C with 10 µg/mL anti-integrin αL, 20 µg/mL anti-integrin β2 (both are subunits of LFA-1), 5 µg/mL anti-integrin α4 (subunit of VLA-4) or 25 µg/mL L-selectin. Simultaneously, the recipient HEC-LTTs were either left untreated or were treated with 25 µg/mL anti-E-selectin, 25 µg/mL anti-VCAM-1 or 10 µg/mL anti-ICAM-1. Pretreated PMNs were then incubated with untreated HEC-LTTs, and untreated PMNs were incubated with pretreated HEC-LTTs for 3 h at 37 °C so that the effect of each antibody against an adhesion molecule was addressed individually. After incubation, PMNs were removed from the recipient cells. On the next day, cultures were fixed and stained for viral IE Ag via indirect immunofluorescence (pink nuclei). Cell nuclei were counterstained with DAPI (purple nuclei). (**A**) Representative exemplary images for recipient HEC-LTTs after PMN-mediated transfer in the presence of function-blocking antibodies. (**B**) The transfer efficiency in the presence of each antibody was calculated and is shown relative to the transfer efficiency of untreated cells. Error bars represent the standard error of the mean of three individual experiments. Asterisks indicate significant differences as compared to the untreated control (* *p*-value < 0.05).

Previous studies suggest that cell-cell contacts between leukocytes and endothelial cells are required for uptake of HCMV into PMNs and subsequent transfer of virus from PMNs to recipient cells [13,19,46]. Therefore, the contribution of the following cellular adhesion molecules to the hematogenous spread of HCMV was investigated: LFA-1 subunits αL and β2, VLA-4 subunit α4, L-selectin (all expressed on PMNs); ICAM-1, VCAM-1, E-selectin (all expressed on endothelial cells). These receptors and ligands enable cell-cell contacts through binding and could thus allow the formation of virological synapses between the cell types (Figure 6). HFFs infected with eight different clinical HCMV isolates were used as donor cultures for the transfer experiments. The cell-associated character of each isolate was verified by incubating the cell culture supernatant overnight with uninfected HFFs. The next day, cultures were fixed and stained for viral IE Ag by indirect immunofluorescence. Isolates were considered to grow strictly cell-associated if no more than 10 IE Ag-positive cells per 15,000 cells were detected (Figure 7). PMNs were freshly isolated as described before and applied to the clinical isolates for 3 h at 37 °C. After recollecting, PMNs were either left untreated or were treated with antibodies against LFA-1 subunits αL or β2, VLA-4 subunit α4 or L-selectin for 30 min at 37 °C. At the same time, the cell culture medium of the recipient endothelial cells was replaced by either fresh medium or medium containing antibodies against ICAM-1, VCAM-1, or E-selectin. Following preincubation, PMNs were applied to the recipient cells for 3 h at 37 °C to allow for virus transfer. PMNs were removed, and the recipient cultures were incubated overnight. Cells were fixed on the next day and stained for viral IE Ag via indirect immunofluorescence. Nuclei were counterstained with DAPI (Figure 8A). Transfer efficiency in the presence of each function-blocking antibody was determined as the percentage of IE Ag-positive cells compared with the total cell count and normalized to untreated transfer (Figure 8B). Each antibody was tested regarding its effect on three different clinical isolates. Blocking of the LFA-1 subunits αL and β2 reduced the transfer efficiency significantly by 40% and 50% (*p*-values < 0.05). Blocking of the VLA-4 subunit α4 and L-selectin did not significantly reduce the transfer. None of the antibodies directed against the adhesion molecules ICAM-1, VCAM-1, or E-selectin on endothelial cells reduced PMN-mediated transfer of HCMV isolates. These results suggest that both subunits of LFA-1 are required for an efficient transfer of cell-associated HCMV from infected PMNs to endothelial cells.

### 3.5. Large Entry Inhibitors Can Reduce PMN-Mediated Transfer of HCMV Clinical Isolates When the LFA-1 Subunit β2 Is Blocked

**Figure 9:**
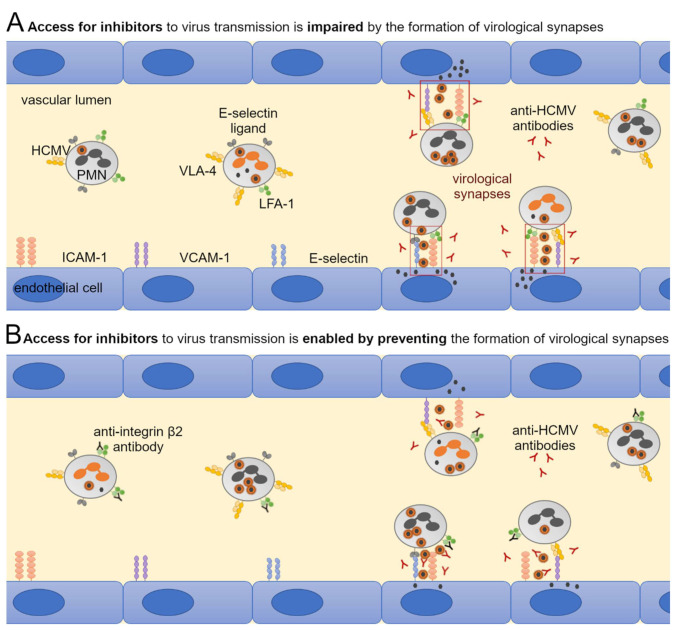
Hypothetical scheme of steric hindrance of neutralizing antibodies by virological synapses and enabled access by the prevented formation of virological synapses. The reaction space between PMN and endothelial cells formed by the binding of cellular adhesion molecules is referred to as a virological synapse. (**A**) As a result of this steric hindrance, large HCMV entry inhibitors such as neutralizing antibodies cannot bind to viral particles that are transferred from PMNs to the endothelial cells and thus cannot prevent infection. (**B**) If the formation of the virological synapse is prevented by function-blocking antibodies to adhesion molecules, the inhibitors can bind to transmitted viral particles and thus reduce infection. Cell and receptor size as well as expression ratios are not shown realistically but are simplified to highlight predicted interactions.

**Figure 10:**
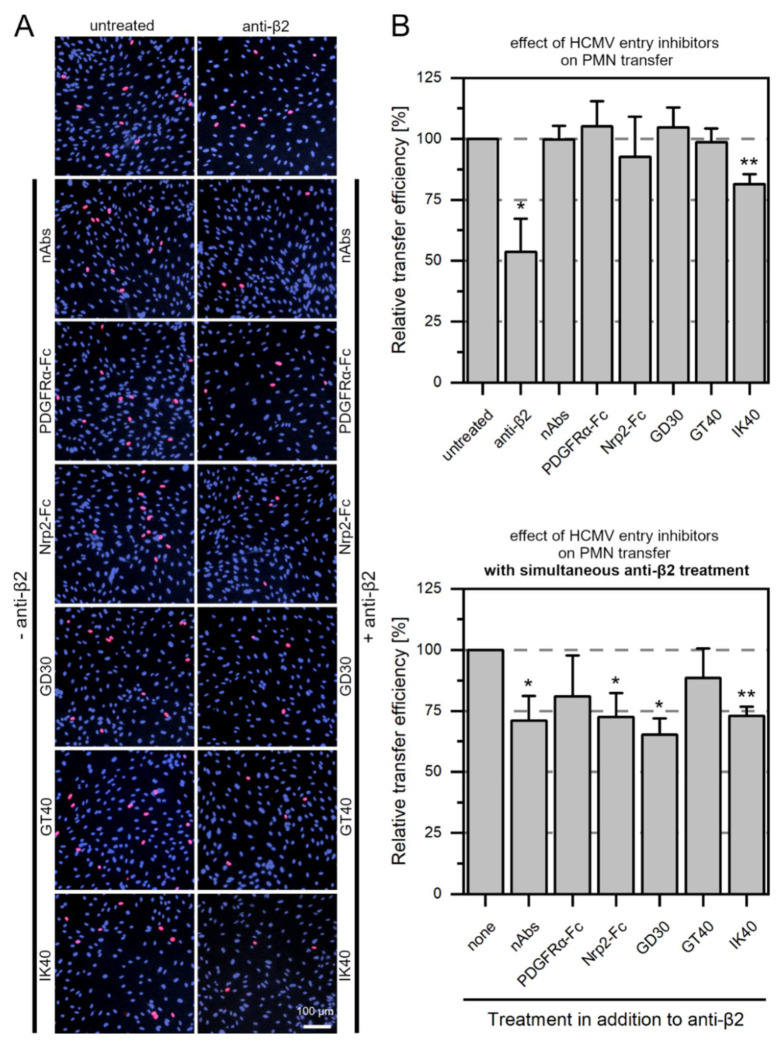
Entry inhibitors reduce PMN-mediated transfer of HCMV isolates to endothelial cells when the β2 subunit of LFA-1 is blocked. PMNs were isolated from EDTA blood of HCMV-seronegative donors and incubated for 3 h at 37 °C with clinical isolate donor cultures. PMNs were recollected and either left untreated or were treated for 30 min at 37 °C with 20 µg/mL anti-integrin β2 (subunit of LFA-1). Then, they were incubated with uninfected recipient HEC-LTTs and HCMV inhibitors for 3 h at 37 °C. HCMV-specific hyperimmunoglobulin (nAbs), recombinant PDGFRα-Fc, recombinant Nrp2-Fc, or the PDGFRα-derived peptides GD30, GT40 and IK40 were added at 0.5 mg/mL, 120 ng/mL, 590 ng/mL or 0.45 mg/mL, respectively. PMNs were removed, and HEC-LTTs were incubated overnight. On the next day, cells were fixed and stained for viral IE Ag via indirect immunofluorescence (pink nuclei). Cell nuclei were counterstained with DAPI (purple nuclei). (**A**) Representative exemplary images for recipient HEC-LTTs after PMN-mediated transfer in the presence of HCMV inhibitors with or without anti-integrin β2 treatment. (**B**) The transfer efficiency in the presence of each inhibitor was calculated and is shown relative to the transfer efficiency of untreated cells (upper graph) or cells treated with anti-integrin β2 only (lower graph). Error bars represent the standard error of the mean of 3 individual experiments. Asterisks indicate significant differences as compared to the untreated control (* *p*-value < 0.05, ** *p*-value < 0.01).

In previous experiments, large entry inhibitors such as neutralizing antibodies or PDGFRα-Fc were ineffective against PMN-mediated transmission of HCMV isolates, whereas smaller 30- and 40-mer peptides inhibited transmission [20]. Virus particles are assumed to be transmitted at reaction spaces created by proximity upon close cell-cell contacts between PMN and endothelial cell, often referred to as a virological synapse. Large entry inhibitors might fail to restrict cell-cell transmission of HCMV as they are hindered from accessing viral particles here (Figure 9A). To test this hypothesis that a virological synapse is formed during PMN-mediated transfer of HCMV, we applied large entry inhibitors (neutralizing antibodies and the soluble receptors PDGFRα-Fc and Nrp2-Fc) or small entry inhibitors (PDGFRα-derived peptides GD30, GT40, and IK40) during incubation of infected PMNs and recipient endothelial cells. In a second approach, the most potent function-blocking antibody from the previous experiments, which targets the LFA-1 subunit β2, was added to the PMNs for a preincubation of 30 min at 37 °C. Then, the same entry inhibitors as before were applied during the incubation of PMNs and endothelial cells. If the hypothetical formation of virological synapses between vehicle PMNs and recipient cells is at least partially reduced by anti-β2, also large entry inhibitors should reduce transfer under this condition (Figure 9B). The next day, cultures were fixed and stained for viral IE Ag via indirect immunofluorescence. Nuclei were counterstained with DAPI (Figure 10A). The transfer efficiency for each treatment with an inhibitor was determined compared to untreated transfer. All inhibitory molecules were evaluated regarding their effect on the same three clinical isolates. As observed in previous experiments [20], neither neutralizing antibodies, PDGFRα-Fc, nor the peptide GT40 were able to inhibit the transfer of cell-associated HCMV via PMNs (Figure 10B). Nrp2-Fc and the 30-mer peptide GD30 also proved ineffective. Hence, IK40 was the only inhibitor that could significantly reduce transfer (*p*-value < 0.01). Incubation of PMNs with only anti-β2 significantly reduced the transfer efficiency by approximately 50% (*p*-value < 0.05), as in the previous experiment. To evaluate the efficacy of the compounds in the presence of anti-β2, the transfer efficiencies of these approaches were normalized to the transfer efficiency of anti-β2 treatment alone. Interestingly, when applied in addition to the function-blocking antibody against β2, all inhibitors showed a reduction of 25% to 30% in addition to the inhibitory effect of anti-β2 alone (Figure 10B). Effects were significant for neutralizing antibodies, Nrp2-Fc, GD30 (*p*-values < 0.05) and IK40 (*p*-value < 0.01). These results suggest that the formation of a virological synapse during PMN-mediated transfer of HCMV may prevent particularly large entry inhibitors from reaching the site of virus transfer between PMN and endothelial cells.

## 4. Discussion

One possible scenario for the spread of HCMV is as follows [59,60,61,62,63,64]: During primary infection, cell-free virus particles initially enter epithelial cells in mucous membranes such as the mouth or gastrointestinal tract. In these tissues, the virus presumably spreads in a cell-associated manner to surrounding cell types such as fibroblasts in connective tissue until it encounters endothelial cells in the blood vessel. There, PMNs take up virus particles from infected endothelial cells and transfer them throughout the bloodstream to uninfected endothelial cells, which then produce progeny of the virus themselves and infect surrounding cell types. Alternatively, dendritic cells are proposed to spread the virus in a cell-associated manner to lymph nodes, which may also promote systemic infection. Glandular tissue might finally secrete cell-free virus particles into body fluids such as breast milk or saliva, transmitting the virus to a new host. While the entry of cell-free HCMV into epithelial and endothelial cells or fibroblasts is quite well understood, it is mostly unclear how PMNs take up the virus and subsequently transfer it to endothelial cells. The gH/gL/gO complex (trimer) and the gH/gL/pUL128-131A complex (pentamer) mediate entry of cell-free virus together with the fusion protein gB [25,26,27,28,65,66]. The trimer binds its cellular receptor PDGFRα on fibroblasts and also appears to be required for infection of other cell types [32,33,34,67]. Recently, Nrp2 was identified as a cellular receptor of the pentamer on endothelial and epithelial cells [29,31]. Although hematogenous spread is assumed to contribute markedly to the pathogenesis of HCMV, molecular mechanisms underlying this type of spread are not as well understood as for entry of cell-free virus. In addition to the time factor and the variety of target tissues that can be subsequently infected, PMN-mediated transmission might offer even more advantages for the virus: On the one hand, the spatial proximity created by rolling adhesion of PMNs to endothelial cells and tight binding of adhesion molecules between the two cell types might offer the possibility that a high dose of infectious virus particles is transmitted. On the other hand, it can be assumed that the virus spreads at shielded sites between PMN and endothelial cells. So far, such a kind of virological synapse has been proposed mainly for the transmission of HIV-1 [40,41]. There is evidence that such a synapse is also formed during hematogenous dissemination of HCMV. Blocking LFA-1 subunits was reported to reduce HCMV uptake to PMNs and subsequent transfer to fibroblasts [19,46]. In addition, HCMV entry inhibitors such as neutralizing antibodies or the soluble decoy receptor PDGFRα-Fc proved ineffective against PMN-mediated transmission, whereas a smaller PDGFRα-derived peptide reduced the uptake and transfer [20]. Assuming that access to sites of virus transmission is denied for large inhibitors, we hypothesized that if blocking LFA-1 would disrupt synapse formation, also large entry inhibitors should neutralize transferred viral particles. The reduced binding between PMN and endothelial cell should allow also neutralizing antibodies or soluble receptor derivatives to reach the transmitted viral particles and thus interfere with transfer of HCMV. Indeed, when LFA-1 was blocked, the large entry inhibitors reduced transfer to a similar extent as the smaller peptides. Considering these findings, inhibitors that are smaller than neutralizing antibodies could be of particular importance for possible antiviral strategies specifically against the hematogenous spread of HCMV. Blocking cellular key players such as adhesion molecules as targets would rather harm the patient, as they play a central and crucial role in a variety of physiological reactions, such as cell-cell stability in tissues or communication between cells in immunological processes such as antigen presentation. The use of small molecule inhibitors, on the other hand, to target PMN-mediated transmission may be considered. Single chain variable fragment (scFv) antibodies may have the advantage over conventional neutralizing antibodies of being able to achieve viral transmission between PMN and endothelial cell. Such antibodies have recently been increasingly tested against various viruses such as hepatitis C virus or measles virus [68,69] and there are reports that they can inhibit the cell-associated spread of HIV-1 in contrast to conventional neutralizing antibodies [70]. scFv have also been studied in the context of HCMV, for example against subunits of gB or the pentamer [71,72], and their use may thus also be promising against PMN-mediated transmission.

Hematogenous dissemination within leukocytes is likely an efficient mode of spread for HCMV: The virus is assumed to spread rapidly throughout the body via blood vessels, infect various cell types starting from endothelial cells, and escape neutralizing antibodies. Our cell culture model offers the opportunity to investigate the dissemination of HCMV via PMNs in detail, with respect to both uptakes into PMNs and entry into the recipient endothelial cells and to identify individual interaction partners. We used stop mutants of individual viral genes in the background of the cell-associated strain Merlin to investigate their contribution to transfer. The pentamer seems to be essential for HCMV uptake into PMNs, as no pp65 was detected in PMNs after incubation with Merlin-RL13tetO-UL128stop. Similar observations were obtained with another HCMV strain [25]. The conclusion that the lack of pp65 signals indicates greatly reduced virus uptake is supported by the finding that transfer of infection by these PMNs was reduced to a similar extent. Whether the trimer also contributes to hematogenous spread has not yet been addressed before, although it is proposed to play a central role in entry into various other cell types, including fibroblasts, epithelial and endothelial cells [67]. Both virus uptake into PMNs and the subsequent infection of endothelial cells was not altered in the absence of gO. Of note, the same stop mutant also showed unaltered focal growth in fibroblast monolayer cultures as long as the pentameric complex was expressed [49], whereas knockdown of UL74 in recent clinical HCMV isolates significantly reduced focus size in this cell type [73]. Hence, we cannot exclude the possibility that the knockout of UL74 in the context of other virus strains may also affect PMN-mediated HCMV transmission. On the other hand, the assumption that the pentamer but not the trimer is involved in PMN-mediated transmission is further supported by the observation that Nrp2-Fc but not PDGFRα-Fc significantly reduced transfer of recent isolates when LFA-1 was blocked.

From a pathophysiologic perspective, cell-associated transmission, whether within a tissue type or via the bloodstream, can greatly facilitate viral spread within the patient, particularly when cell-free virus is neutralized by antibodies. The assumption that HCMV requires the cell-free transmission route primarily to infect a new host, whereas spread within the host occurs mainly in a cell-associated manner, is also supported by studies in the murine cytomegalovirus (MCMV) model, where gO was required for primary replication after virus injection but dispensable for the subsequent spread in most tissues [64,74]. In a similar study with a more physiological route of infection via the respiratory tract, slight variations of this theme were observed [75]. Primary infection of lung epithelial cells also depended on gO, whereas systemic cell-associated spread via infected myeloid cells was initially intact in the MCMV mutant without gO. However, over time, cell-associated transmission via myeloid cells also decreased, probably because lung epithelial cells inefficiently replicated the virus and could not serve as a source for further uptake by myeloid cells. If this also applies to HCMV, gO-mediated enhancement of replication in fibroblasts may indirectly contribute to PMN-mediated dissemination. To address this hypothesis in the future, BAC-clones of those recent HCMV isolates are desirable, in which a contribution of gO to cell-associated spread in fibroblasts has been found. 

The mutational approach provided insights into a possible role of tegument proteins pp65 and pp71 in hematogenous spread. While already the uptake of HCMV into PMNs was significantly reduced for the pp71 stop mutant, the effect was even more pronounced for the subsequent transfer to endothelial cells. Strong growth deficits have been reported for cell-free growth of pp71-deleted viruses [76,77,78]. This protein is assumed to have a functional role besides the transactivation of the IE proteins 1 and 2, as co-transfection experiments demonstrated its involvement in efficient infection of surrounding cells [79]. Consistent with this, our pp71 stop mutant showed a reduced focus size in fibroblast monolayers (Supplementary Figure S2). Significantly less virus was also transferred to endothelial cells from PMNs that have been infected with the pp65 stop mutant. In previous experiments with a pp65-deleted virus, this protein was attributed the role of a scaffold protein responsible for the proper packaging of viral proteins into the outer tegument and thus for the assembly of functional virions [80]. This function could be responsible for the observed phenotypes in the stop mutants. Although such data have not been collected for pp71, it is tempting to speculate that this abundant tegument protein also either has a direct effect on virion packaging or, at least, its absence might result in improperly packaged virus particles. Thus, the strong effect on PMN-mediated transmission of HCMV could be due to the fact that in the absence of pp65 or pp71, virions are either more poorly packaged or even released in lower numbers as mature virus particles from the infected donor cultures. During focal growth of the mutants in fibroblast monolayers, this circumstance might be overcome by the high multiplicity of infection that is observed for the cell-associated spread of HCMV [35,37]. Since we have demonstrated that the tegument proteins pp65 and pp71 contribute to the pathophysiologically relevant dissemination via leukocytes, the use of one of these proteins as a potential candidate for a future HCMV vaccine might be even more approachable. A pp65- or pp71-deleted virus as a candidate for vaccine development could offer the advantage of still being able to spread locally and slow in tissues but having a major deficit in terms of fast and efficient transmission via the bloodstream. Initial attempts to develop such vaccines have already been reported for other CMVs: An attenuated deletion vaccine of the pp65 homolog GP83 was tested in the guinea pig model and showed significantly reduced placental infection as well as reduced neonatal mortality compared to the placebo-treated cohort [81]. Concerning RhCMV, an Rh100-deleted vector vaccine prevented transmission from mother to child and via leukocyte transfusion. This protein shows a homology of 41% to pp71 of HCMV [18].

In summary, our mutational approach indicates that the trimer does not contribute to the uptake of HCMV into PMNs and further transfer to endothelial cells, whereas the pentamer is required for this transmission process which is regarded as crucial for hematogenous dissemination. The viral tegument protein pp65 appears to be critically involved in virus spread via leukocytes, which may be exploited for developing an HCMV vaccine that is specifically attenuated concerning systemic spread. In comparison, the loss of pp71 obviously causes a more general attenuation. In addition, our data support the idea that the binding of adhesion molecules between PMNs and endothelial cells establishes a virological synapse that presumably shields large entry inhibitors from sites of HCMV transmission. Therefore, small compound inhibitors may be promising candidates to interfere with this step during hematogenous spread.

## Figures and Tables

**Table 1:** Primers used for the generation of mutant BAC genomes.

Primer	Sequence (5′-3′)
*Merlin-UL82stop for*	GTCTCAGGCATCGTCCTCGCCCGGTGAGGGACCCTCGTCGTAAGCAGCAGCAATTAGTTAAGCCGAAGCCGCCAGCGGAAGaggatgacgacgataagt
*Merlin-UL82stop rev*	GGCAGTGCAGGCGACCAAAGCTTCCGCTGGCGGCTTCGGCTTAACTAATTGCTGCTGCTTACGACGAGGGTCCCTCACCGGcaaccaattaaccaattctga
*Merlin-UL82stop-REV for*	GTCTCAGGCATCGTCCTCGCCCGGTGAGGGACCCTCGTCAGAAGCAGCAGCAATTAGTGAAGCAGAAGCCGCCAGCGGAAGaggatgacgacgataagt
*Merlin-UL82stop-REV rev*	GGCAGTGCAGGCGACCAAAGCTTCCGCTGGCGGCTTCTGCTTCACTAATTGCTGCTGCTTCTGACGAGGGTCCCTCACCGGcaaccaattaaccaattctga
*Merlin-UL82stop short for*	GTCTCAGGCATCGTCCTCGC
*Merlin-UL83stop for*	CGCAGGCAGCATGGAGTCGCGCGGTCGCCGTTGTCCAGAATAGATATCCGTACTGTGACCCATTTCGGGGCACGTGCTaggatgacgacgataagtaggg
*Merlin-UL83stop rev*	CGCGACTAAACACTGCTTTCAGCACGTGCCCCGAAATGGGTCACAGTACGGATATCTATTCTGGACAACGGCGACCGCcaaccaattaaccaattctgattag
*Merlin-UL83stop-REV for*	CGCAGGCAGCATGGAGTCGCGCGGTCGCCGTTGTCCAGAAATGATATCCGTACTTGGTCCTATTTCGGGGCACGTGCTaggatgacgacgataagtaggg
*Merlin-UL83stop-REV rev*	CGCGACTAAACACGGCTTTCAGCACGTGCCCCGAAATAGGACCAAGTACGGATATCATTTCTGGACAACGGCGACCGCcaaccaattaaccaattctgattag
*Merlin-UL83stop short for*	CGCAGGCAGCATGGAGTCGC
*repMerlin-UL83stop for*	CGCAGGCAGCATGGAGTCGCGCGGTCGCCGTTGTCC**C**GAATAGATATCCGTACTGTGACCCATTTCGGGGCACGTGCTaggatgacgacgataagt
*repMerlin-UL83stop1 rev*	TCACAGTACGGATATCTATTC**G**GGACAACGGCGACCGCcaaccaattaaccaattctga
*repMerlin-UL83stop2 rev*	CGCGACTAAACAC**G**GCTTTCAGCACGTGCCCCGAAATGGGTCACAGTACGGATATCTA
*repMerlin-UL83stopREV1 for*	ATGATATCCGTACT**G**GGTCC**C**ATTTCGGGGCACGTGCTaggatgacgacgataagt
*repMerlin-UL83stopREV2 for*	CGCAGGCAGCATGGAGTCGCGCGGTCGCCGTTGTCC**C**GAAATGATATCCGTACT**G**GGT
*repMerlin-UL83stopREV1 rev*	ACC**C**AGTACGGATATCATTTC**G**GGACAACGGCGACCGCcaaccaattaaccaattctga
*repMerlin-UL83stopREV2 rev*	CGCGACTAAACACGGCTTTCAGCACGTGCCCCGAAAT**G**GGACC**C**AGTACGGATATCAT
*Kanamycin universal rev*	CAACCAATTAACCAATTCTGA

Note: primer sites encoding mutated codons are underlined; uppercase letters: HCMV sequence; lowercase letters: Kanamycin cassette; grey letters: silent mutations to overcome potential hairpin formation; bold: repair of silent mutations at potential hairpin sites.

## Data Availability

Not applicable.

## Supplementary Materials

The following supporting information can be downloaded at: https://www.mdpi.com/article/10.3390/v14071561/s1. Figure S1: Comparison of Merlin-RL13tetO wild-type and the original revertant Merlin-RL13tetO-UL83stop-REV during PMN-mediated transfer. Figure S2: Focal growth of Merlin-RL13tetO wild-type, Merlin-RL13tetO-UL83stop and Merlin-RL13tetO-UL83stop-REV, and Merlin-RL13tetO-UL82stop and Merlin-RL13tetO-UL82stop-REV in fibroblast monolayers. Table S1: Sequence analysis of mutants Merlin-RL13tetO-UL83stop and Merlin-RL13tetO-UL83stop-REV. Figure S3: Characterization of mutants.
