# Supplementary material for: Viral and Cellular Factors Contributing to the Hematogenous Dissemination of Human Cytomegalovirus via Polymorphonuclear Leukocytes
Source: Viruses. 2022 Jul 18;14(7):1561. doi: 10.3390/v14071561 (PMC9323586; doi:10.3390/v14071561)
Supplement: Supplementary file 1 [file viruses-14-01561-s001.zip › viruses-1737099-supplementary.pdf]

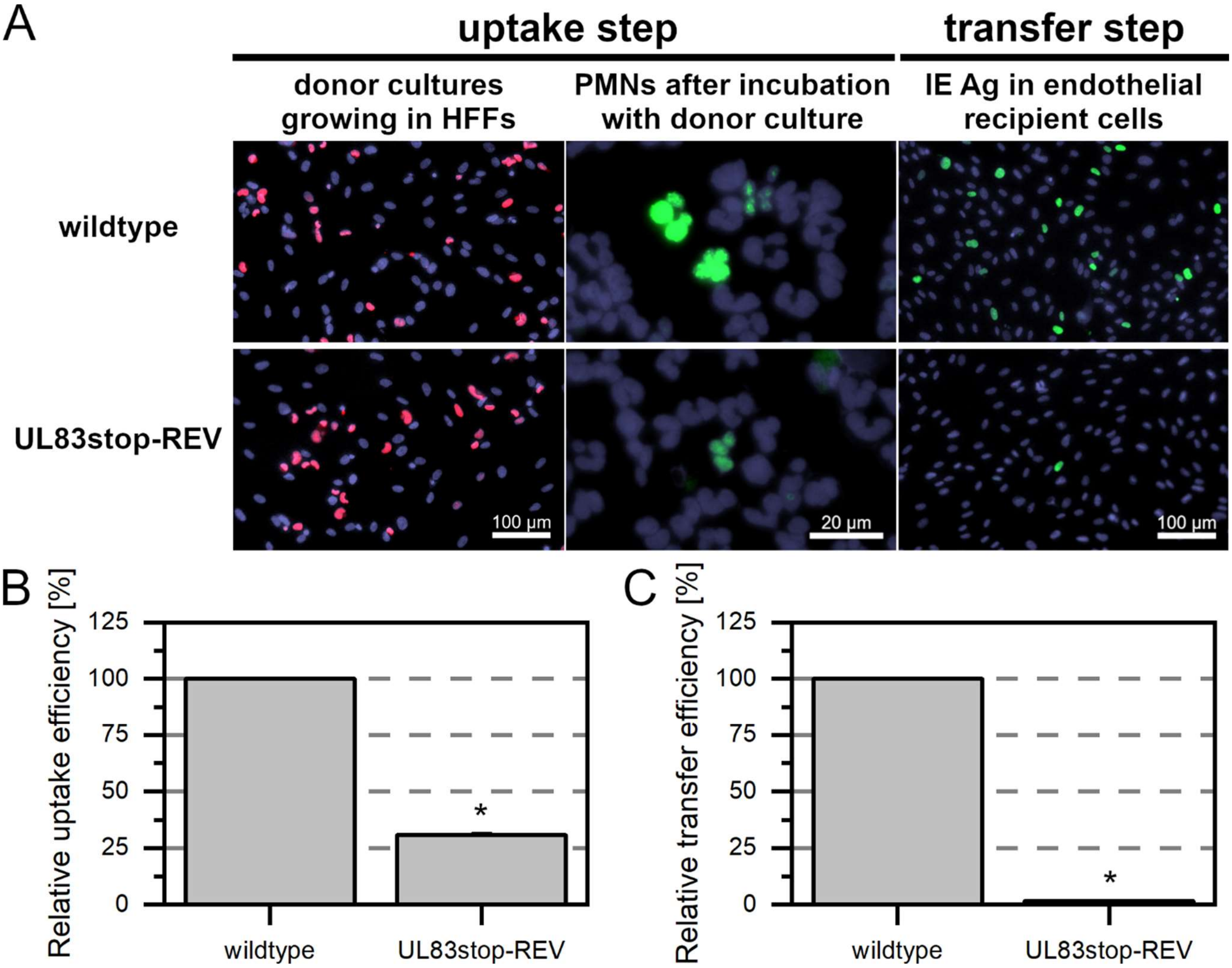

Figure S1. Comparison of Merlin-RL13tetO wildtype and the original revertant Merlin-RL13tetO-UL83stop-REV during PMN-mediated transfer. A PMNs were isolated from EDTA blood of HCMV-seronegative donors and incubated for 3 h at 37 °C with Merlin-RL13tetO or Merlin-RL13tetO-UL83stop-REV. PMNs were recollected, and a fraction was used for preparation of cytospots. Donor cultures were fixed and stained via indirect immunofluorescence for viral IE Ag (pink nuclei). PMNs were fixed and stained for viral pp65 (green nuclei). The remaining PMNs were incubated with uninfected recipient HEC-LTTs for 3 h at 37 °C. After incubation, PMNs were removed. On the next day, cultures were fixed and stained for viral IE Ag via indirect immunofluorescence (green nuclei). Cell nuclei were counterstained with DAPI (purple nuclei). **B** The uptake efficiency was calculated as the fraction of pp65-positive PMNs compared to all PMNs and was normalized to uptake of wildtype Merlin-RL13tetO. Bars indicate mean values of 4 individual experiments, error bars represent the standard error of the mean (SEM). **C** The transfer efficiency was calculated as the fraction of IE Ag-positive cells compared to the overall cell count and was normalized to PMN-mediated transfer of wildtype Merlin-RL13tetO. Bars indicate mean values of 4 individual experiments, error bars represent the SEM.

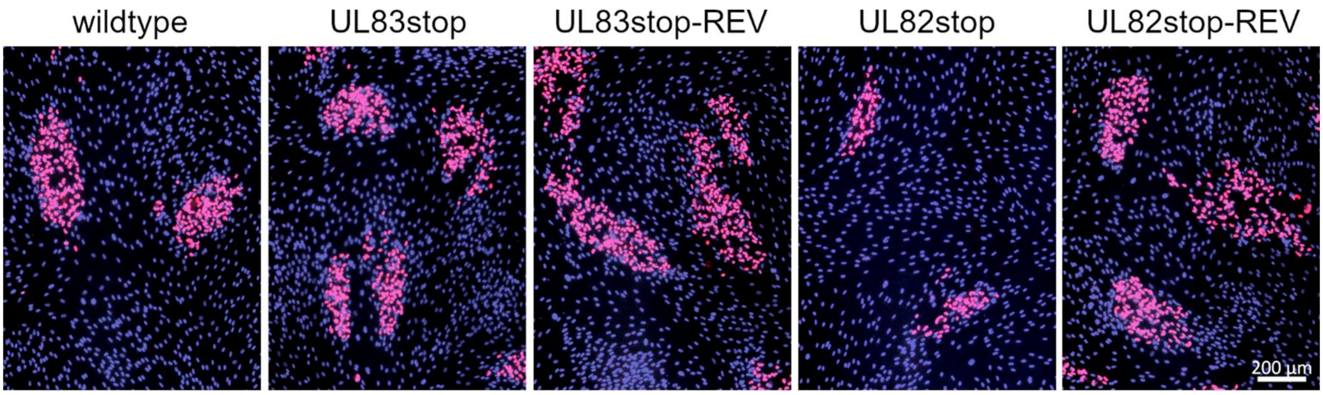

Figure S2. Focal growth of Merlin-RL13tetO wildtype, Merlin-RL13tetO-UL83stop and Merlin-RL13tetO-UL83stop-REV, and Merlin-RL13tetO-UL82stop and Merlin-RL13tetO-UL82stop-REV in fibroblast monolayers. HFFs, which were infected with wildtype virus or the respective mutants were co-cultured with uninfected HFFs for 7 days. Cultures were fixed and stained via indirect immunofluorescence for viral IE Ag (pink nuclei). Cell nuclei were counterstained with DAPI (purple nuclei).

Table S3. Sequence analysis of mutants Merlin-RL13tetO-UL83stop and Merlin-RL13tetO-UL83stop-REV. BAC-DNAs used to reconstitute the respective viruses were subjected to whole-genome analysis on an Illumina MiSeq System with the NEB Next Ultra II FS DNA PCRfree library Kit. Using the Geneious Prime software, individual paired reads were assembled to a modified Merlin reference genome (derived from GenBank #GU179001) containing the tet operator sequence upstream of the RL13 ORF. Table represents results from variant calling of each assembly as compared to the reference genome.

sample	CDS	codon number	Codon change	translation	Protein effect
Merlin-RL13tetO-UL83stop	UL83	9	CCC > CCA	P P	no
	UL83	11	ATG > TAG	M *	truncation
	UL83	16	GGT > TGA	G *	truncation
Merlin-RL13tetO-UL83stop-REV	UL83	9	CCC > CCA	P P	no
	UL83	15	CTG > CTT	L L	no
	UL83	17	CCC > CCT	P P	no

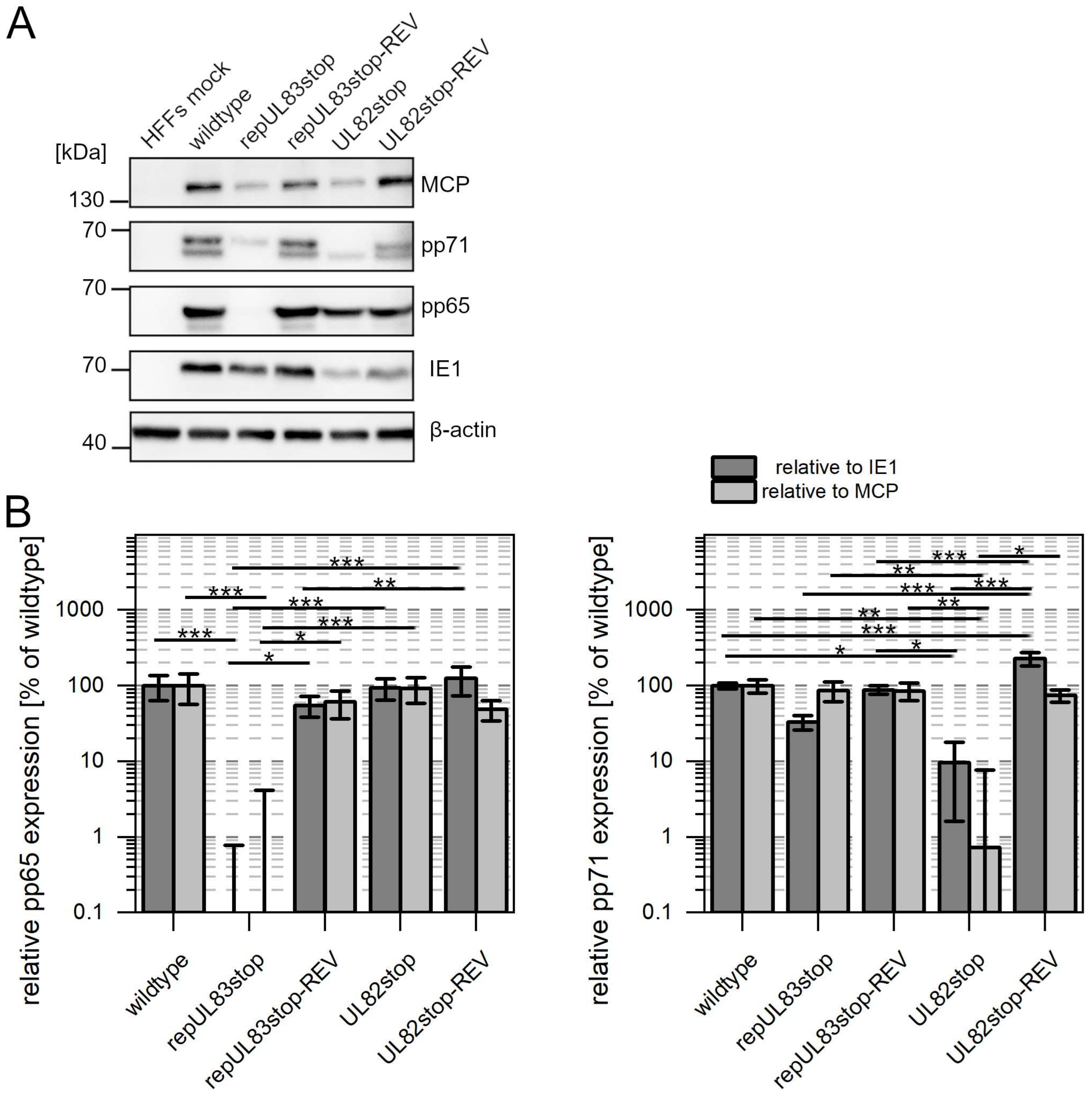

Figure S4. Characterization of mutants. A Lysates of uninfected or Merlin-infected HFFs were analyzed by separation on a 10% polyacrylamide gel under reducing conditions. Immunoblotting was performed using antibodies against viral pp71 (UL82), pp65 (UL83), IE1 (UL123), MCP (UL86) and cellular β-actin. Numbers on the left side indicate the molecular mass in kDa. **B** For Merlin wildtype and UL83 or UL82 stop mutants and revertants thereof, the ratio of pp65/IE1, pp65/MCP, pp71/IE1 and pp71/MCP signals were determined to evaluate the respective protein expression. Bars indicate mean values of eight replicates (two blots of four independent lysate preparations). Error bars represent the standard error of the mean (SEM). Asterisks indicate significant differences (*, $p < 0.05$; **, $p < 0.01$; ***, $p < 0.001$).
